# A Novel Pipeline for Drug Repurposing for Bladder Cancer Based on Patients’ Omics Signatures

**DOI:** 10.3390/cancers12123519

**Published:** 2020-11-26

**Authors:** Marika Mokou, Vasiliki Lygirou, Ioanna Angelioudaki, Nikolaos Paschalidis, Rafael Stroggilos, Maria Frantzi, Agnieszka Latosinska, Aristotelis Bamias, Michèle J. Hoffmann, Harald Mischak, Antonia Vlahou

**Affiliations:** 1 Biotechnology Division, Biomedical Research Foundation of the Academy of Athens, 11527 Athens, Greece; mmokou@bioacademy.gr (M.M.); vlygirou@bioacademy.gr (V.L.); ioangel@med.uoa.gr (I.A.); rstrog@bioacademy.gr (R.S.); 2 Mosaiques Diagnostics GmbH, 30659 Hannover, Germany; frantzi@mosaiques-diagnostics.com (M.F.); latosinska@mosaiques-diagnostics.com (A.L.); mischak@mosaiques-diagnostics.com (H.M.); 3 Cellular Immunology Laboratory, Center for Basic Research, Biomedical Research Foundation of the Academy of Athens, 11527 Athens, Greece; npaschal@bioacademy.gr; 4 Haematology-Oncology Unit, Department of Clinical Therapeutics, Alexandra Hospital, National and Kapodistrian University of Athens, 11528 Athens, Greece; abamias@med.uoa.gr; 5 Department of Urology, Medical Faculty, Heinrich-Heine-University Düsseldorf, 40225 Düsseldorf, Germany; michele.hoffmann@hhu.de; 6 British Heart Foundation Glasgow Cardiovascular Research Centre, University of Glasgow, Glasgow G12 8QQ, UK

**Keywords:** bladder cancer, drug repurposing, CMap, omics, proteomic, signature, NMIBC subtypes, MIBC, urothelial cell carcinoma cell lines, mTOR inhibitors

## Abstract

**Simple Summary:**

Current therapies for bladder cancer present limitations as, regularly, not all patients benefit from treatment. Drug repurposing refers to the use of available drugs in new disease contexts. In this study, we identified drugs of potential to be repurposed, e.g., used in the treatment of aggressive bladder cancer. This was achieved by using earlier described molecular profiles (mRNAs, proteins) of tissue of patients with bladder cancer, matched to molecular profiles induced by drugs via mathematical tools. Through this process, multiple drugs potentially treating aggressive bladder cancer were predicted. Of these, an inhibitor of the mTOR molecular pathway was further investigated and found to significantly delay the growth of many bladder cancer cell lines. Collectively, this study provides a robust pipeline for drug repurposing based on tissue molecular profiles and highlights drug candidates meriting further experimental investigation in the treatment of aggressive bladder cancer.

**Abstract:**

Multi-omics signatures of patients with bladder cancer (BC) can guide the identification of known de-risked therapeutic compounds through drug repurposing, an approach not extensively explored yet. In this study, we target drug repurposing in the context of BC, driven by tissue omics signatures. To identify compounds that can reverse aggressive high-risk Non-Muscle Invasive BC (NMIBC) to less aggressive low-risk molecular subtypes, the next generation Connectivity Map (CMap) was employed using as input previously published proteomics and transcriptomics respective signatures. Among the identified compounds, the ATP-competitive inhibitor of mTOR, WYE-354, showed a consistently very high score for reversing the aggressive BC molecular signatures. WYE-354 impact was assessed in a panel of eight multi-origin BC cell lines and included impaired colony growth and proliferation rate without any impact on apoptosis. Overall, with this study we introduce a promising pipeline for the repurposing of drugs for BC treatment, based on patients’ omics signatures.

## 1. Introduction

Bladder cancer (BC) is the 10th most common cancer worldwide [1] with Non-Muscle Invasive BC (NMIBC) accounting for approximately 70% of new BC cases and Muscle Invasive (MIBC) representing the remaining 30% [2]. Based on clinicopathological characteristics stratification of NMIBC to subgroups of low-, intermediate- and high- risk for progression is applied [3]. For the former, the standard of care is transurethral resection of the bladder tumor (TURBT), whereas for intermediate- and high-risk NMIBCs, TURBT is followed by intravesical Bacillus Calmette-Guérin (BCG) immunotherapy or chemotherapy for one to three years due to the substantial risk of recurrence and progression to MIBC [3]. Nevertheless, almost 40% of the NMIBC patients fail to respond to first-line BCG therapy [4]. In these cases, radical cystectomy is recommended [3]. For MIBC, cisplatin-based chemotherapy and cystectomy remain the standard first-line treatment, but relapse rates remain high for stages >pT2 or pN+ [5]. Within the last five years several immune checkpoint inhibitors have been assessed in the treatment of advanced urothelial carcinoma with five agents eventually receiving approval by the U.S. Food and Drug Administration (FDA) as novel therapies in specific disease settings [6]. Nevertheless, only a subset of patients benefit with long-term durable responses to such treatments, while discontinuation of therapy may be required due to immune-related adverse events [7]. Considering the need for repeated endoscopic assessments and resections, especially in high-grade lesions, and the increasing number of patients who require more intensive therapy, BC is among the most costly malignancies on a per-patient basis [8,9]. For the above reasons, better disease management for MIBC as well as high-risk NMIBC in a personalized and cost-effective manner is urgently needed.

A cost-effective strategy towards new cancer therapies accelerating clinical translation is drug repurposing. The approach targets the reuse of existing de-risked drugs for new therapeutic purposes [10,11]. In such a strategy, repurposing hypotheses can be established by combining machine-learning computational methods with chemical structure, genotype, large-scale transcriptomics and proteomics data [10]. Several resources have been introduced in recent years for data-driven drug repurposing with the Connectivity Map (CMap) being among the most well-known tools [12]. CMap was first released in 2006 [13], and was recently massively scaled up as part of the NIH’s LINCS program to generate a large-scale compendium of gene expression signatures of human cells’ response to chemical and genetic perturbations using the L1000 gene expression platform [14]. CMap relies on the signature reversion principle based on which drugs that have the potential to reverse in silico the expression profile of a given set of hallmark genes for a particular disease likely have therapeutic potential for that disease [10,13,14]. CMap has been successfully applied for the identification of repurposed drugs in a wide range of diseases [15,16,17] but, to the best of our knowledge, a CMap-based drug repurposing approach has not been applied in BC yet.

In this study, we propose a drug repurposing approach driven by molecular signatures of patients with BC, using the CMap resource, based on previously generated/published omics signatures. In a recent publication, comprehensive tissue proteomic analysis enabled the stratification of NMIBC patients into three groups, revealing a continuum of proteome changes from less to more aggressive NMIBC and eventually MIBC [18]. In brief, three distinct non-invasive proteomic subtypes were identified with NPS1 representing high-stage/-grade/-risk cancers and NPS3 mostly low-stage/-grade/-risk cancers [18]. Specifically, these molecular/proteomic subtypes correlated well with the pathologic classification, with NPS1 harboring mostly T1-grade 3-high risk tumors and NPS3 containing mostly Ta-grade 1-low risk tumors (based on EORTC classification) [3,18]. Along these lines, NPS3 tumors presented a differentiated/luminal molecular phenotype whereas NPS1 tumors were rich in basal markers [18]. Several observed changes at the protein expression level between these two groups overlapped with respective changes at the transcriptome level derived in part from the same patients, but also from published independent datasets from NMIBC progressors in comparison to non-progressors [18,19,20]. In the study presented here, these molecular changes and data were used in a CMap analysis aiming to retrieve a list of candidate drugs that could potentially reverse (at least parts of) the molecular signature of aggressive NMIBC. The in vitro impact of the top candidate in the context of the disease was additionally verified using a panel of multi-origin BC cell lines.

## 2. Results

### 2.1. Identification of Drugs with Reversal Potential for High-Risk NMIBC

**Figure 1:**
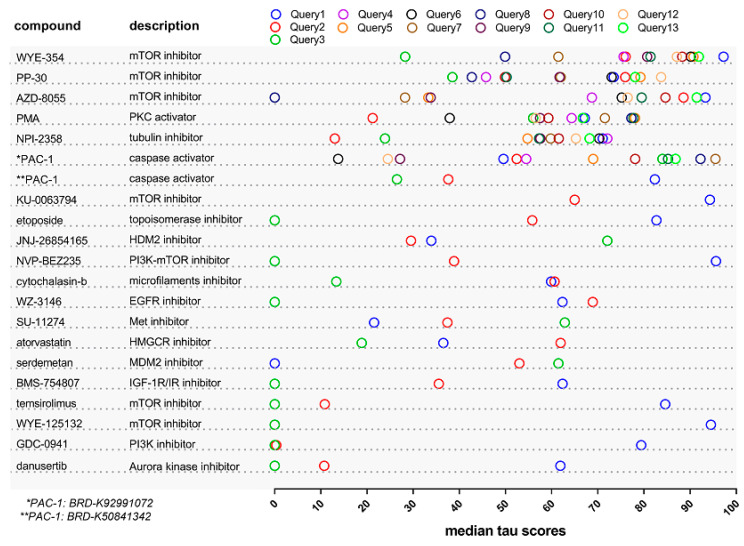
Compounds that can potentially reverse the proteomic signature of high-risk Non-Muscle Invasive bladder cancer (NMIBC) (NPS1; [18]) to low-risk NMIBC (NPS3; [18]). For the CMap analysis the differentially expressed proteins in NPS3/NPS1 [18] were used as input. Queries 1–3 were performed using subsets of 150 proteins ranked based on their fold change (as presented in Table S1) whereas for Queries 4–13 randomized sublists were used as input (as presented in Table S2). The listed compounds include only those that had a median tau score more than 60 in at least one of the three queries for Queries 1–3 or in more than six queries for Queries 4–13. The full list of retrieved compounds is presented in Table S3. Compounds with the same name (e.g., PAC-1) are presented more than one time since they correspond to different BRD IDs (BRD ID, or Broad ID, corresponds to an identifier that was developed by the Broad Institute to uniquely address a particular perturbagen).

To identify known de-risked compounds for the treatment of aggressive BC (defined as high-risk NMIBC) the next generation CMap [14] was employed. Proteomic features characterizing the high-risk NMIBC (NPS1) subgroup from our previously published high-resolution tissue proteomics analyses [18] were initially utilized as input data in the CMap tool, as described in Methods. Given the limit of the CMap tool to a maximum of 150 features as input dataset, multiple queries were performed using different subsets of proteins characterizing this group (Tables S1 and S2). Among the retrieved compounds (Table S3) only those with a positive connectivity score predicted to reverse the high-risk non-muscle invasive signature (thus, reversing the aggressive phenotype), were considered when interpreting the results. As shown in Figure 1, six compounds were repetitively identified from the independent queries at highest relative scores, of which three corresponded to mTOR inhibitors (WYE-354, PP-30 and AZD-8055), one to the tubulin inhibitor NPI-2358, the PKC activator phorbol-12-myristate-13-acetate (PMA), and the caspase activator PAC-1 (Figure 1, Table S4). Among these six compounds WYE-354, a specific ATP-competitive inhibitor of mTOR, was ranked top, presenting the highest average median tau score and was prioritized as a candidate to potentially reverse the aggressive NMIBC phenotype.

**Figure 2:**
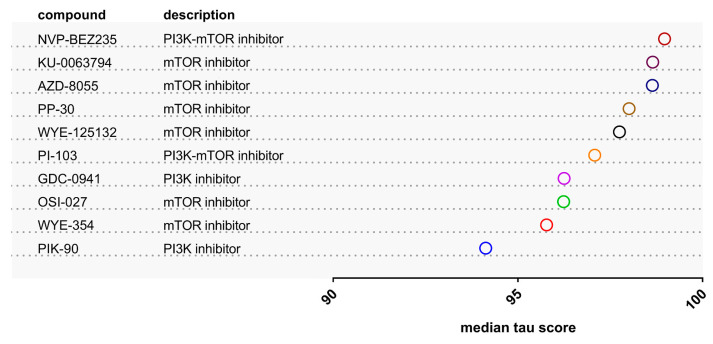
The ten compounds with the highest median tau score across the nine core cell lines of CMap that may reverse transcriptomics and proteomics features associated with aggressive BC phenotypes (explanations provided in the main text and in Table S6).

To further investigate the validity of the prediction, a signature of 116 differentially expressed proteins between NMIBC and MIBC, overlapping with the low-risk NMIBC (NPS3) distinctive features [18] and with concordant expression at the mRNA level in low- vs high-risk subtype comparisons in previously published transcriptomic studies [19,20] was used as input in the CMap analysis (Table S5; for details on these proteins please see Table S6 from Stroggilos et al. [18]). Among the retrieved compounds (Table S6) with a positive median tau score (reversing the aggressive BC signature), ten compounds were shortlisted as having connectivity scores >90. These belonged to the classes of PI3K and mTOR inhibitors (Figure 2). Interestingly, WYE-354, PP-30, and AZD-8055 were overlapping with the six retrieved compounds during the initial analysis (Figure 1). In both cases, the high score of WYE-354 indicated that this may be a promising compound for BC therapy since it may reverse both MIBC and aggressive phenotype of NMIBC to early NMIBC stages. Although the rest two mTOR inhibitors may also be very promising anti-neoplastic compounds, WYE-354 was carried forward for further detailed investigation in vitro as a result of its availability.

### 2.2. The Effect of the WYE-354 mTOR Inhibitor on BC Cell Lines In Vitro

#### 2.2.1. WYE-354 Reduces the Growth of BC Cells

**Figure 3:**
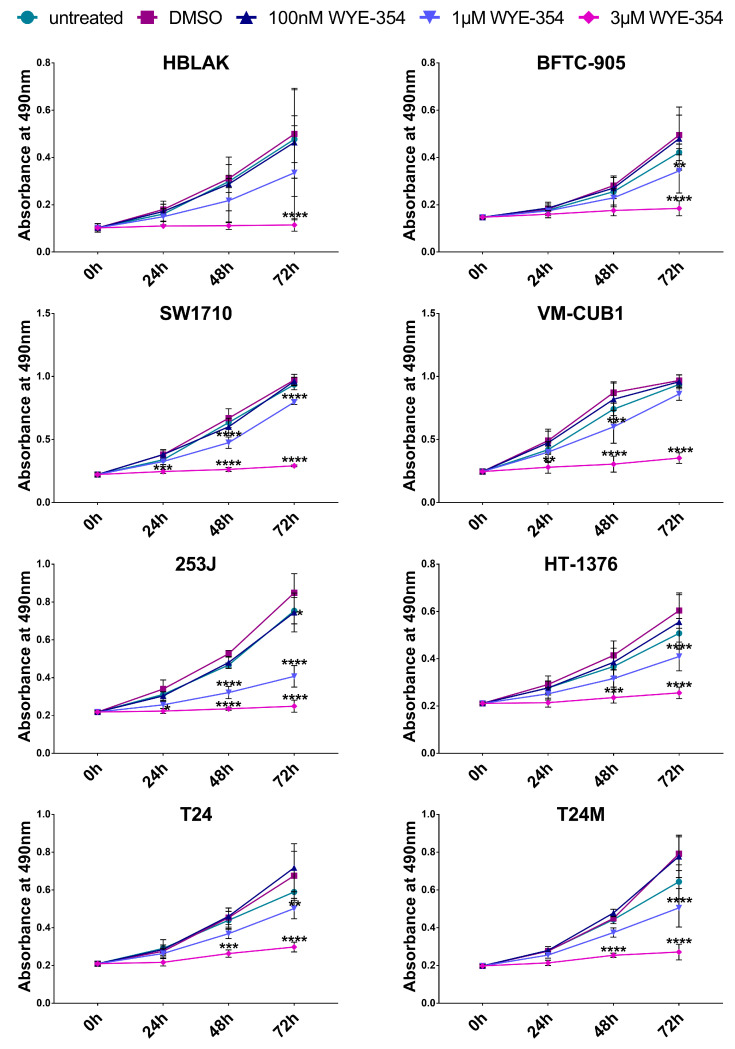
The impact of the WYE-354 inhibitor on a panel of multi-origin bladder cancer (BC) cell lines using the MTS cell proliferation assay. The line graphs represent the absorbance (OD) at 490 nm of BC cells untreated as well as treated with 0.1% Dimethyl sulfoxide (DMSO), 100 nM, 1 μM or 3 μM WYE-354 at three different time points (24, 48 and 72 h). DMSO-treated cells were used as control. The values represent the means ± SD from three independent experiments performed in five replicates each time. Statistical significance was determined using the two-way ANOVA test. Statistical significant differences between the treated with the inhibitor and the DMSO-treated cells in each time point are indicated (* *p* < 0.05, ** *p* < 0.01, *** *p* < 0.001, **** *p* < 0.0001).

To elucidate the possible impact of the WYE-354 mTOR inhibitor onto the malignant phenotype of BC cells in vitro, a panel of multi-origin BC cell lines, including benign (HBLAK), non-muscle invasive (BFTC-905, SW1710) and muscle invasive (T24, T24M, VM-CUB1, 253J, HT-1376) cells, were employed. The above cell lines were subjected to treatment with WYE-354 at different concentrations (100 nM, 1 μM, and 3 μM) and the impact was examined on cell proliferation using the MTS assay at different time points (24, 48, and 72 h). As shown in Figure 3, WYE-354 administration resulted in a significant reduction on the proliferation rate of all the tested BC cell lines in a concentration-dependent manner. Specifically, treatment with 1 μM and 3 μM of the WYE-354 inhibitor significantly reduced the proliferation rate of BC cells in comparison to the Dimethyl sulfoxide (DMSO)-treated cells at each time point investigated (Figure 3).

**Figure 4:**
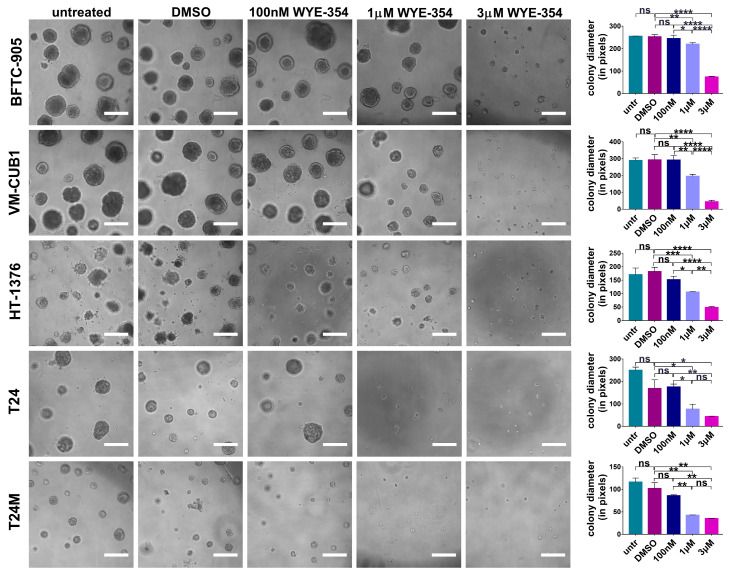
The effect of WYE-354 on the growth of BC cells in 3D cultures. Representative images of the colonies formed by the BC cells after 11 days of growth in matrigel either without treatment or treated with 0.1% DMSO, 100 nM, 1 μM or 3 μM WYE-354. DMSO-treated cells were used as control. Results from untreated cells are also shown. Magnification: 10×. The bar graphs on the right side represent the average length of the colonies formed by the BC cells under the tested conditions. The values represent the means ± SD from three independent experiments performed in triplicate each time. Statistical significance was determined using the one-way ANOVA test (* *p* < 0.05, ** *p* < 0.01, *** *p* < 0.001, **** *p* < 0.0001, ns: not significant). Scale bars: 500 μm.

The impact of the WYE-354 inhibitor was also investigated when the cells were grown in 3D cultures in matrigel. As illustrated in Figure 4, the BC cells that were subjected to treatment with 1 μM and 3 μM WYE-354 presented a substantial decrease in the diameter of the colonies in comparison to those treated with DMSO. At lower concentrations of the inhibitor (at 100 nM), no significant impact could be observed (Figure 4). In addition, in all tested cell lines no significant impact on the number of colonies was observed. It should be also noted that for the HBLAK, SW-1710 and 253J BC cell lines the assay was not applicable since the cells were not able to form colonies under these experimental conditions. Collectively, these findings indicate impairment in growth which is in concordance with the findings from the cell proliferation assay.

#### 2.2.2. WYE-354 Does Not Affect BC Cell Viability

**Figure 5:**
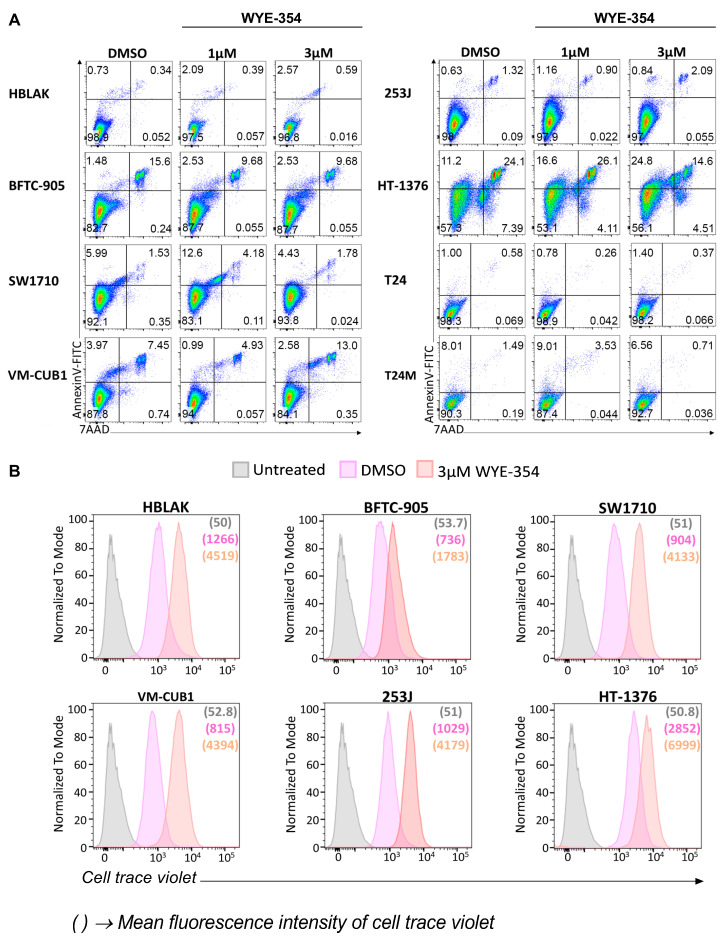
The impact of WYE-354 on cell viability. (**A**) Apoptosis and necrosis were examined by flow cytometry analysis of Annexin V and 7-AAD staining in BC cells treated for 48 h with 0.1% DMSO, 1 μM or 3 μM WYE-354. DMSO-treated cells were used as control. (**B**) Cell viability and proliferation was followed for 72 h using the CellTrace™ Violet reagent for a panel of representative BC cell lines. The discrete peaks for DMSO and 3 μM WYE-354 in the histograms represent the live proliferating cells. The untreated and unlabeled cells were used as controls for the flow cytometry analysis and are indicated in purple. DMSO-treated cells were used as control for the analysis of the impact of WYE-354. The numbers in brackets represent the mean fluorescence intensity of the cell trace violet stain within the cells.

To further examine the impact of WYE-354 on apoptosis, BC cells were treated with different concentrations of the inhibitor (1 μM, 3 μM) for 48 h and subsequently analyzed by flow cytometry for apoptotic (via Annexin-V staining) and necrotic (via 7-aminoactinomycin D (7-AAD) staining) cells. As shown in Figure 5A, no significant effect was observed in cell apoptosis/necrosis upon treatment with WYE-354 at different concentrations in comparison to the DMSO-treated cells, also demonstrating the absence of non-specific toxic effects of the inhibitor. To trace back the viable cells with flow cytometry, a subset of representative BC cells was initially labeled with cell trace violet and subsequently treated with DMSO or 3 μM WYE-354 for 72 h (Figure 5B). Although, no discrete generations could be observed because of the broad staining intensity distribution, the distinct peaks in the histograms indicate that the cells remain labeled and, thus, viable 72 h after the treatment (Figure 5B). Furthermore, the shift in the cell populations between the two conditions (DMSO and 3 μM inhibitor) (Figure 5B) indicate a delayed proliferation rate in the presence of the inhibitor, which is in agreement with the findings from the MTS and colony formation assay. Overall, it becomes evident that the WYE-354 mTOR inhibitor has a significant impact on BC cell growth likely via delaying proliferation without affecting cell viability.

## 3. Discussion

Despite the significant advances in BC therapy, the heterogeneity of the disease and the limitations of the current therapeutic options necessitate the exploitation of alternative strategies. Drug repurposing is considered a promising approach [21], accelerating transfer to the clinic and reducing costs in comparison to de novo drug discovery [22]. In the context of BC, drug repurposing was adopted for use of cationic amphiphilic drugs, a class of commonly used antidepressants, antihistamines and antipsychotics drugs, that demonstrated an antineoplastic efficacy in BC cell lines, in a human orthotopic BC xenograft model and ex vivo in patient-derived tissues [23]. In this case, repurposing was based on evaluation of retrospective/observational studies providing evidence that the incidence of BC in patients with schizophrenia was significantly lower in comparison to individuals without schizophrenia [23]. Along the same lines, the repurposing potential of Nitazoxanide, a cysticidal drug with immuno-anticarcinogenic action, was investigated in a NMIBC animal model and showed therapeutic potential when used simultaneously with BCG treatment [24]. A high-throughput screening using a library of known chemicals and drugs identified disulfiram as a repurposed drug that enhances sensitivity to cisplatin in BC [25]. Furthermore, the efficacy of a wide range of drugs selected based on their clinical relevance for BC (e.g., assessed in clinical trials, targeting relevant pathways and molecules of interest) was evaluated in BC organoid cell lines highlighting the impact of trametinib and gemcitabine in vitro in organoid cultures and in vivo in orthotopic xenografts [26]. In our study, we report on a novel pipeline that can be applied for drug repurposing in BC using molecular signature-based computational approaches and patients’ omics data followed by in vitro evaluation of the compounds therapeutic potential in a panel of BC cell lines.

CMap is a commonly used computational approach that has been applied in several investigations for drug repurposing by comparing disease and drug induced molecular features based on gene expression data [12]. CMap analysis is based on data at the transcriptome level which constitutes a limitation of the approach when using as input other omics (such as proteomics, as in our case) data. Nevertheless, large scale quantitative proteomics data have been previously employed successfully for CMap analysis in the context of complex diseases, such as diabetic kidney disease [15] and cardiovascular disease [16], resulting in the identification of promising therapeutic drugs. In our case, to address as possible this limitation, one of our queries used as input a distinctive signature of 116 differentially expressed features with concordant mRNA and protein expression levels in low- vs high-risk NMIBC subtypes; and consistency in the predictions among the independent queries was used to shortlist candidates. Furthermore, considering that CMap is based on the expression of 978 “landmark” genes with the expression levels of the 81% of non-measured transcripts being inferred computationally, an increased probability of false discovery may be expected. Thus, for the interpretation and prioritization of the retrieved compounds not only the connectivity scores should be considered (ideally from multiple complementary analyses as was the case of our described multiple queries), but an extensive literature mining and search in drug databases/resources should be performed. Another limitation of CMap is that not all cancer types are represented; for example, in the nine core cell lines profiled in the resource, no BC cell lines are included; which further emphasizes the need for experimental verification of the predictions. To the best of our knowledge a signature-based drug repurposing strategy using high resolution omics datasets has not been applied in BC yet.

In our study, CMap analysis using proteomic signatures supported also by expression differences at the transcriptomics level, predicted several promising compounds that may potentially reverse aggressive high-risk NMIBC or MIBC molecular phenotypes. Several mTOR inhibitors (WYE-354, AZD-8055, PP-30) were predicted among the top ranked compounds. The identification of mTOR inhibitors as top candidates is expected, considering that the phosphoinositide 3 kinase (PI3K)/ protein kinase B (AKT)/mammalian target of rapamycin (mTOR) pathway is a key driver of carcinogenesis and progression in BC [27]. Genetic alterations, such as mutations, copy number alterations or RNA expression changes in the PI3K/AKT/mTOR pathway are present in more than 40% of BCs [28]. Thus, the predicted therapeutic potential of the mTOR inhibitors in BC further supports the validity of our pipeline. Another perturbagen class with predicted potential for aggressive NMIBC was the caspase activator PAC-1, a small scaffold procaspase activator, with a promising anticancer action based both on in vitro and in vivo models [29]. PAC-1 was also described to induce apoptosis in combination with different FDA-approved chemotherapeutics across many cancer types and chemotherapeutic targets, and is currently in phase I clinical trials (NCT02355535) [29]. This evidence is in line with the predicted therapeutic potential from our CMap analysis, suggesting that this compound may also merit further evaluation. Along the same lines, the tubulin inhibitor NPI-2358, a tubulin-depolymerizing agent, appears to be another promising candidate since it has a potential in vitro antitumor activity and it acts as a tumor vascular disrupting agent, being also tested in phase I clinical trials [30,31]. At first sight, apparently contradictory to the predictions from our CMap analysis, the PKC activator PMA acts as tumor promoter in cancer and is also implicated in BC progression, with existing studies supporting that PKC inhibition may be useful for treating metastatic BC [32,33] and increasing the chemosensitivity of BC cells [34]. However, the tumor promoting effect of PMA is indirect, via its ability to induce PKC dependent terminal differentiation [35]. This process leads to the positive selection of cells not properly responding to the signal. These cells will continue to proliferate, while all others terminally differentiate. In other words, the tumor-promoting effect of PMA is the result of (likely mutant) cells not responding correctly to the stimulus PKC activation. In the approach presented here, the impact of PMA is predicted to initiate (terminal) differentiation in the BC cells, and given the fact that cancer cell de-differentiation is a hallmark of cancer [36], treatment with PMA would most likely be beneficial. Based on these considerations, the CMap prediction appears reasonable. In support of this hypothesis, it should be noted that PKC inhibitors have in general not been proven successful in clinical trials with current studies reconsidering the PKC role in cancer [37].

Among the top-ranked compounds were the ATP-competitive mTOR inhibitors WYE-354, PP-30 and AZD-8055 that target the active sites of both mTORC1 and mTORC2 [38]. The use of mTOR inhibitors for BC has been tested with limited success [39], with the mTORC1 inhibitor everolimus possessing a meaningful antitumor activity only in a subset of patients with advanced BC [40]. The advantage of ATP-competitive mTOR inhibitors (such as the presented WYE-354) is that they inhibit the PI3K/AKT/mTOR signaling cascade both upstream and downstream of AKT, resulting in more complete mTOR blockade and consequently more potent activity than inhibition of mTORC1 alone [41]. Along these lines, existing evidence also supports that dual mTOR inhibitors (Torin-2 and KU-0063794) may have anti-proliferative effects in BC cell lines that exhibit high phosphorylation of AKT (Ser-473) [42]. In our analysis, the WYE-354 inhibitor was ranked top among the predicted drugs. WYE-354 is a potent and specific ATP-competitive mTOR inhibitor that blocks substrate phosphorylation by mTORC1 (P-S6K(T389)) and mTORC2 (P-AKT(S473)), with significant selectivity for mTOR over PI3Kα (>100-fold) and PI3Kγ (>500-fold) [43]. The therapeutic potential of WYE-354 has been previously highlighted in the context of cancer further supporting the predicted potential of the compound and the validity of our repurposing pipeline approach. WYE-354 has been reported to induce cell cycle and proliferation arrest as well as apoptosis and down-regulation of angiogenic factors in a diverse set of cancer cell lines including gliomas, breast, colorectal and prostate cancers [43]. In vivo studies demonstrated that WYE-354 exerts a strong antitumor activity in mouse models of prostate, gliomas [43] and hepatocellular carcinomas [44]. Further, a combination of WYE-354 with standard chemotherapy was reported to sensitize cancer cells to overcome multidrug resistance in acute myeloid leukemia [45].

In our study, the WYE-354 impact was assessed in a panel of eight multi-origin BC cell lines. For six of the tested cell lines (BFTC-905, SW1710, VM-CUB1, 253J, HT-1376 and T24) mutational data that were available at the Cancer Cell Line Encyclopedia (CCLE) portal (https://portals.broadinstitute.org/ccle) confirmed the activated status of the mTOR signaling pathway and indicated no interference of mutations in genes involved in the mTOR signaling pathway and thus in the WYE-354 mechanism of action (Table S7). WYE-354 administration in BC cells significantly attenuated the proliferation of the cells which is in concordance with the observed impact in other cancer types. However, no significant impact on cells’ apoptosis could be detected. Our in vitro results suggest an impact of the compound in highly aggressive (represented by the invasive cell lines used) but also less aggressive (BFTC-905, SW1710) cells. As part of a recent study of pooled-cell line chemical-perturbation viability screens for 4518 compounds against 578 human cancer cell lines using the PRISM method, WYE-354 was screened in a panel of 23 BC cell lines (including BFTC-905, 253J, SW1710, HT-1376, VM-CUB1, and T24) [46]. In this study, an impact on BC cell viability in response to WYE-354 was observed in concentrations higher than 1 μM for 253J, BFTC-905, SW1710 and HT-1376 as illustrated in the DepMap portal (https://depmap.org/portal/). However, in those cell lines a concentration gradient dependency was not obvious in all the replicates and additionally the impact of WYE-354 for VM-CUB1 and T24 was inconclusive. The observed discrepancies may be explained by the different applied methods; drug response in a pool of different cell lines in their study [46] instead of detailed single cell line viability profiling at different time points in our study. For the accurate quantification of the drug impact, further in vivo studies are required to also evaluate the drug effect on the surrounding stroma, including the infiltrating immune cells, whose interplay with the epithelial cells will ultimately confer the final observed impact on the cancer.

Our study has certain limitations. We did not include normal adjacent tissues, which restricts the CMap analysis towards the prediction of compounds that may reverse the MIBC or high-risk NMIBC to low-risk NMIBC and not to the healthy state. Another limitation is the lack of transcriptomic/proteomic data after treatment with WYE-354 that would support the reversal of the molecular signature. Such data may help to mechanistically explain the observed impact of the drug. In addition, our observations are based on in vitro studies and no preclinical studies were performed. Further evaluation of the identified compounds in animal BC models is required to support the reliability of the suggested pipeline. Although WYE-354 showed significant antiproliferative effects in all tested BC cell lines, the changes detected in the benign HBLAK cells suggest that systemic administration may result in adverse effects in normal non-cancerous tissues. Finally, this study focused solely on the evaluation of the therapeutic efficacy of WYE-354. However, as mentioned above, a significant number of other promising compounds, not restricted to mTOR inhibitors, were retrieved following our drug repurposing pipeline that merit further investigation; while use of combinations of drugs should be also considered in future studies. Nevertheless, to the best of our knowledge this is the first omics-driven drug repurposing strategy in BC that is based on patient profiling data.

## 4. Materials and Methods

### 4.1. Connectivity Map Analysis

For the next generation CMap analysis (https://clue.io/) [14], gene expression signatures were created using our previously published [18] proteomics data from patients with NMIBC at low (NPS3) and high-risk (NPS1). These included 223 significantly up- and 297 significantly down- regulated proteins as defined by the ratio NPS3/NPS1. Due to the technical limit of a maximum of 150 genes that can be uploaded in the CMap platform, and in order to cover the whole list of differentially regulated features, the initial gene lists were divided into different subsets of 150 genes either based on the fold change (Queries 1–3, Table S1) or based on a randomization strategy (Queries 4–13, Table S2). The different lists of up- and down-regulated proteins were submitted as gene names in the “Query” of CMap tools, against the Touchstone reference dataset of gene expression (L1000) to directly explore the similarity (or dissimilarity) of the query data with the reference perturbagen signatures. The retrieved perturbagens were ranked according to the CMap connectivity score (median tau score) that ranges from −100 to +100 and displayed as a heat map of the connected compounds. Positive scores indicated similarity between the compound’s signature and that of the query while negative scores demonstrated an opposing relationship of the two signatures. The absolute values of the connectivity scores corresponded to the magnitude of the correlation of the perturbagen to the query with the higher ones to be considered as strong scores for generating hypotheses. Due to the fact that the averages of the median tau scores were below 70 among the different queries and considering that a high score (ideally above 90) is more accurate, the following criterion was set to increase the validity of the findings; only compounds presenting with a tau score >60 in at least one query (for Queries 1–3) or in more than six queries (for Queries 4–13) were considered for further analysis (Table S3). For increasing validity of predictions and shortlisting compounds, further CMap analyses using as input a key signature of 116 features from aggressive NMIBC with concordant expression patterns at the protein level in MIBC based on our previously published proteomics data [18] and concordant expression patterns at the mRNA level when comparing progressors vs non-progressors from the UROMOL and LUND transcriptomic studies [19,20] were performed. (Table S5, for details please refer to Table S6 from Stroggilos et al. [18]).

### 4.2. Cell Culture

The BC cell lines BFTC-905, SW1710, VM-CUB1, 253J, and HT-1376 were from the Department of Urology, Medical Faculty, Heinrich-Heine-University Düsseldorf, Düsseldorf, Germany. T24 human bladder carcinoma cells were obtained from American Type Culture Collection (ATCC). The T24M human bladder cancer cell line represents a metastatic variant of T24 cells that was previously established [47] and reflects malformations encountered in aggressive BC. All cell lines were cultured in DMEM (Gibco-BRL, Paisley, Scotland, UK) supplemented with 10% fetal bovine serum (FBS; Gibco-BRL, Paisley, Scotland, UK) and 1% Penicillin-Streptomycin (Pen-Strep; Gibco-Invitrogen) at 37 °C in 5% CO_2_ under sterile conditions. The immortalized uroepithelial cell line HBLAK that was previously characterized as an in vitro urothelial cancer cell model [48] was obtained from CELLnTEC (CELLnTEC, Bern, Switzerland) and was cultured according to the manufacturer’s instructions in serum-free CnT-Prime Epithelial Culture Medium (CELLnTEC, Bern, Switzerland) at 37 °C in 5% CO_2_.

### 4.3. WYE-354 Inhibitor

For the inhibition experiments the WYE-354 ATP-competitive inhibitor of mTOR (Selleckchem, Houston, TX, USA) was added in the growth medium of the BC cells at 100 nM, 1 μM, and 3 μM. 0.1% DMSO-treated cells were used as controls since DMSO was the solvent of the inhibitor.

### 4.4. MTS Cell Proliferation Assay

The effect of the mTOR inhibitor WYE-354 on the proliferation rate of the BC cell lines was assessed using the MTS cell proliferation assay. Cells were seeded in 96-well plates at a density of 1000 cells per well or 3000 for HBLAK and were subsequently subjected to 0.1% DMSO, 100 nM, 1 μM or 3 μM WYE-354 and cultured for 24, 48 and 72 h. Untreated cells were also included. DMSO-treated cells were used as control. The proliferation rate of the cells was assessed after adding the recommended amount of the MTS reagent (Promega, Madison, WI, USA) at the indicated time points. Following an incubation of 3 h at 37 °C in a humidified, 5% CO_2_ atmosphere the absorbance was then recorded at 490 nm with a 96-well plate reader (SPECTROstar Nano, BMG LABTECH). At least three independent experiments were performed with each experiment containing five replicates.

### 4.5. Colony Formation Assay

The impact of the mTOR inhibitor WYE-354 on the colony forming ability of the BC cell lines was assessed using 96-well plates coated with 50 μL of growth factor-reduced Matrigel (BD Biosciences, Franklin Lakes, NJ, USA). Cells were plated at a density of 1000 cells per well and were treated with 0.1% DMSO, 100 nM, 1 μM or 3 μM WYE-354. Untreated cells were also analyzed. DMSO-treated cells were used as control. The plates were incubated for 11 days at 37 °C in a humidified, 5% CO_2_ atmosphere. Photographs from five different fields (10×) per well were taken using a Leica CTR MIC microscope. The number and the size of the colonies were analyzed by using the Image J software (version 1.51j8). Three independent experiments were performed with each experiment including at least three replicates. For the BC cell lines HBLAK, SW1710 and 253J the assay was not applicable.

### 4.6. Apoptosis Analysis

The FITC Annexin V Apoptosis Detection Kit with 7-aminoactinomycin D (7-AAD) (BioLegend, San Diego, CA, USA) was performed according to manufacturer’s recommendations to investigate BC cell apoptosis and necrosis. Cells were treated with 0.1% DMSO, 1 μM, 3 μM WYE-354 or remained untreated for 48 h and subsequently stained according to the manufacturer’s instructions. DMSO-treated cells were used as control. The cells were analyzed by flow Cytometry using the FACS-ARIA-III (Becton Dickinson Biosciences, Franklin Lakes, NJ, USA). All plots were selected for singlet live cells according to FSC/SSC and FSC-H/FSC-A parameters. Data analysis was performed with FlowJo software (Becton Dickinson Biosciences, Franklin Lakes, NJ, USA).

### 4.7. Cell Trace

The CellTrace™ Violet Cell Proliferation Kit, for flow cytometry (Thermo Fisher Scientific, Waltham, MA, USA) was performed according to manufacturer’s recommendations to determine the proliferation of BC cell populations upon treatment with the WYE-354 inhibitor. BC cells were stained with 1 μM CellTrace Violet for 20 min at 37 °C. After removing the dye and washing the cells, the cells were subsequently subjected to treatment with DMSO or 3 μM WYE-354. After 3 days, the fluorescence intensities of the cell populations were analyzed by flow cytometry using the FACS-ARIA-III (Becton Dickinson Biosciences, Franklin Lakes, NJ, USA). The untreated and unlabeled cells were used as controls for the flow cytometry analysis. WYE-354 treated cells were compared to DMSO-treated cells. Data analysis was performed with FlowJo software.

### 4.8. Statistical Analysis

For multiple group analyses, the one-way and two-way ANOVA tests were performed followed by a Tukey post hoc test for the colony formation and the MTS assay respectively. Data are presented as mean ±SD (* *p* < 0.05, ** *p* < 0.01, *** *p* < 0.001, **** *p* < 0.0001, ns: not significant). All statistical analyses were performed using GraphPad Prism 7 software (GraphPad Software, San Diego, CA, USA).

## 5. Conclusions

In this study, we introduce a promising pipeline to form drug repurposing hypotheses in BC based on patients’ omics signatures. Such a strategy has not been explored yet. In the proposed pipeline a computational-based approach using the newly released version of the CMap resource is applied. We investigated proteomic signatures according to the different molecular subtypes of the disease, placing emphasis on features characterizing aggressive phenotypes (aggressive NMIBC). Cross-omics (transcriptomics and proteomics) data were also employed to increase the validity of the approach and facilitate shortlisting of the compounds. In line with the existing knowledge from other cancer types, our repurposing approach predicted several drugs that may have antineoplastic impact in BC. WYE-354, a dual mTOR inhibitor, presented antitumor effect in a panel of BC cell lines, further supporting the potential of our proposed pipeline. Investigation of other retrieved candidates or combinations thereof is currently pursued including further evaluation of their therapeutic efficacy in preclinical animal models.

## Supplementary Materials

The following are available online at https://www.mdpi.com/2072-6694/12/12/3519/s1, Table S1: The differentially expressed proteins in NPS3/NPS1 that were used as input for the CMap analysis in Queries 1–3; Table S2: A randomization approach to develop ten different sublists of the differentially expressed proteins in NPS3/NPS1 that were used as input for the CMap analysis in Queries 4–13. The rank based on abundance (shown also in Table S1) is provided next to each protein; Table S3: The retrieved compounds from the CMap analysis of Queries 1–13; Table S4: Perturbagens that can potentially reverse the proteomic signature of high-risk NMIBC (NPS1) to low-risk (NPS3) were present and had a median tau score more than 60 in at least one of the three queries for Queries 1–3 or in more than six queries for Queries 4–13; Table S5: The 116 differentially expressed proteins between NMIBC and MIBC overlapping with NPS3 distinctive features and with also concordant expression at the mRNA level in low- vs. high-risk subtype comparisons in previously published transcriptomic studies [19,20]; Table S6: The retrieved compounds from the CMap analysis of the 116 features presented in Table S5; Table S7: Mutational data of genes implicated in the mTOR pathway that were available at the Cancer Cell Line Encyclopedia (CCLE) portal for the tested BC cells lines.
